# The Role of MicroRNA in the Pathogenesis of Duchenne Muscular Dystrophy

**DOI:** 10.3390/ijms25116108

**Published:** 2024-06-01

**Authors:** Kajetan Kiełbowski, Estera Bakinowska, Grzegorz Procyk, Marta Ziętara, Andrzej Pawlik

**Affiliations:** 1 Department of Physiology, Pomeranian Medical University, 70-111 Szczecin, Poland; kajetan.kielbowski@onet.pl (K.K.); esterabakinowska@gmail.com (E.B.); martazietara44@gmail.com (M.Z.); 2 1st Chair and Department of Cardiology, Medical University of Warsaw, Banacha 1A, 02-097 Warsaw, Poland; grzegorz.procyk@wum.edu.pl; 3 Doctoral School, Medical University of Warsaw, 02-091 Warsaw, Poland

**Keywords:** Duchenne muscular dystrophy, non-coding RNA, microRNA

## Abstract

Duchenne muscular dystrophy (DMD) is an X-linked progressive disorder associated with muscle wasting and degeneration. The disease is caused by mutations in the gene that encodes dystrophin, a protein that links the cytoskeleton with cell membrane proteins. The current treatment methods aim to relieve the symptoms of the disease or partially rescue muscle functionality. However, they are insufficient to suppress disease progression. In recent years, studies have uncovered an important role for non-coding RNAs (ncRNAs) in regulating the progression of numerous diseases. ncRNAs, such as micro-RNAs (miRNAs), bind to their target messenger RNAs (mRNAs) to suppress translation. Understanding the mechanisms involving dysregulated miRNAs can improve diagnosis and suggest novel treatment methods for patients with DMD. This review presents the available evidence on the role of altered expression of miRNAs in the pathogenesis of DMD. We discuss the involvement of these molecules in the processes associated with muscle physiology and DMD-associated cardiomyopathy.

## 1. Introduction

Duchenne muscular dystrophy (DMD) is a severe disorder characterised by progressive muscular impairment [1]. The disease usually begins when a child starts to walk [2], and the early symptoms include difficulties with climbing stairs, muscle weakness, frequent falls, bone fractures, scoliosis, and a delay in achieving motor milestones [3]. Furthermore, patients experience symptoms in other systems due to muscle weakness. For example, deteriorating respiratory muscles may lead to complications such as pneumonia, mucus plugging, atelectasis, and even respiratory failure [4]. Consequently, the disease leads to rapid loss of ambulation and a need for assisted ventilation. In recent years, the life expectancy of patients with DMD has improved. Nevertheless, respiratory or cardiac insufficiency is significantly associated with premature death, with an average survival of approximately 28 years for those born after 1990 [5]. Understanding the pathogenesis of DMD is crucial to developing novel treatment methods. For example, a recent study demonstrated the important role of the gut microbiome in the pathogenesis of DMD [6].

Non-coding RNAs (ncRNAs) have been reported to regulate the progression of numerous diseases, including cardiological conditions, autoimmune disorders, and malignancies [7,8,9,10,11]. These molecules mediate gene expression; thus, their dysregulation can significantly contribute to the progression of the disease. Therefore, monitoring ncRNAs can be used in the diagnostic process, and, importantly, manipulation of ncRNA expression may induce beneficial effects. ncRNAs are grouped into several families, including microRNAs (miRNAs), long non-coding RNAs (lncRNAs), and circular RNAs, among others. MiRNAs can be released into the circulation due to cell damage or the active secretion of these molecules in extracellular vesicles [12]. MiRNAs specific to the muscle tissue are known as myomiRs, and their dysregulation in the circulation has been observed in muscular diseases, suggesting altered release of these molecules. In this review, we discuss the available evidence regarding the potential use of miRNAs in the diagnosis of DMD and their involvement in the pathogenesis of the disease.

## 2. Duchenne Muscular Dystrophy: Pathogenesis and Epidemiology

There are a broad number of muscular dystrophies, progressive diseases associated with muscle weakness, that have different genetic backgrounds. The pathogenesis of DMD and Becker dystrophy is associated with mutations in the gene that encodes dystrophin, which is located on the X chromosome [13,14]. The incidence of the disease is estimated at 1 in 3600 male liveborn infants [15]. Dystrophin is expressed in skeletal and cardiac muscle, as well as in the brain, where it is expressed in postsynaptic inhibitory neurons [16,17,18]. It consists of four major domains, including the N-terminus actin-binding domain, a central rod domain, a cysteine-rich domain interacting with the transmembrane protein β-dystroglycan, and a C-terminal domain [19]. The protein links muscle cell cytoskeleton with proteins located in the cell membrane (sarcolemma) [20], and it is involved in the structure known as the dystrophin-associated protein complex (DAPC), which takes part in maintaining muscle cell membrane stability during contraction [21]. Furthermore, dystrophin is involved in the processes of cellular adhesion, morphogenesis of various tissues, and cellular signalling [22,23,24]. Dystrophin represents only 0.002% of total muscle protein, but its deficiency is associated with severe diseases known as dystrophies [25]. These progressive myopathies affect skeletal and cardiac muscles. As the disease progresses, muscle tissue degenerates and is replaced with fibrotic and fatty tissue. Consequently, myocytes become unstable due to sarcolemma deformation and micro-tears, which activate cation channels [26]. Moreover, dystrophin deficiency is associated with an overload of calcium, an ion that acts as a second messenger. As a result, a cascade of inflammatory responses is induced, which causes mitochondria to produce reactive oxygen species (ROS) [27,28]. During early phases, degenerated muscle may be regenerated; however, this process depends on the number of satellite cells [2,27,29,30]. Muscle stress induced by the degenerative processes negatively impacts its functionality, and, therefore, more stressed muscles such as the diaphragm are affected earlier [2,31].

Depending on dystrophin availability and compensatory mechanisms, several phenotypes of dystrophies have been described. For example, the course of Becker muscular dystrophy (BMD) is less severe than that of DMD [2,32,33]. Consequently, establishing a definite diagnosis may be challenging in several cases. To distinguish dystrophinopathies and predict the phenotype, a reading frame hypothesis has been proposed and considered to have great significance [34]. According to this hypothesis, the genetic backgrounds of BMD and DMD differ. In contrast to BMD, the mutations that lead to DMD frequently involve deletions or duplications that disrupt the reading frame, as well as nonsense mutations that result in the formation of unstable dystrophin [2,34]. However, a recent study demonstrated that the predictive value of this hypothesis was 82.5%, lower than expected [35]. Due to the length of the gene encoding dystrophin, it is susceptible to mutations. It is estimated that two-thirds of DMD cases arise due to exon deletion (exons 8–17 and exons 45–53) [36,37,38].

## 3. MicroRNAs in the Pathogenesis of Duchenne Muscular Dystrophy

### 3.1. MicroRNAs Regulating Muscle and Satellite Cells

**Figure 1:**
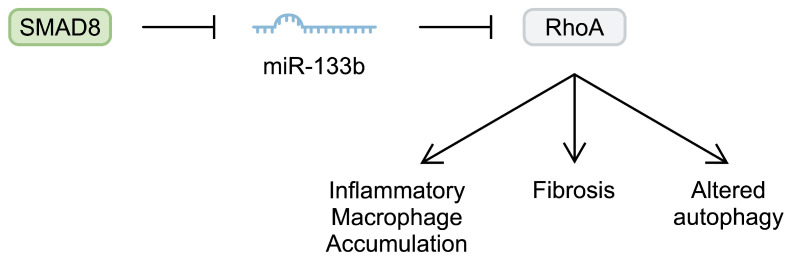
Schematic representation of the Smad8/miR-133b/RhoA pathway that is implicated in the pathogenesis of Duchenne muscular dystrophy.

Non-coding RNAs are frequently dysregulated in inflammatory and neoplastic diseases. Altered expression of ncRNAs changes gene transcription, which then impacts signalling pathways, leading to aberrant cellular behaviours [7,9]. Several studies have examined the role of miRNAs in DMD. MyomiRs are important in muscle physiology, as they regulate skeletal muscle development, cellular proliferation, and differentiation. This group includes miR-1, miR-206, miR-208b, miR-133a and miR-133b, and miR-499 [39]. Altered expression of these myomiRs has been associated with the development of DMD. For example, miR-133b has been implicated in muscle diseases, as its plasma levels are reduced in patients with sarcopenia [39]. miR-133b might slow the progression of DMD. In the mdx mouse model, miR-133b deletion reduced the muscle cross-sectional area (CSA). Moreover, the deletion impaired the myotube formation of satellite cells and reduced muscle regenerative capabilities. Interestingly, deletion of miR-133b might not be an induction factor for the pathogenesis of DMD; rather, it might contribute to exacerbated pathological mechanisms. Specifically, deletion of this miRNA in healthy mice was not associated with broad changes in the transcription profile in healthy mice. By contrast, in mdx mice, its deletion significantly affected several signalling pathways, including the apelin, peroxisome proliferator-activated receptors α (PPARα), and signal transducers and activators of transcription 3 (STAT3) pathways. Furthermore, it induced significant transcriptional alterations, including upregulation of miR-133b targets such as RhoA and transcription factor SP1, among others [40]. Altered expression of these molecules can also contribute to DMD features. For example, RhoA is antimyogenic and antiadipogenic, while it promotes osteogenesis. The use of a RhoA inhibitor in a dystrophin/utrophin double-knockout mouse has been associated with improved myogenic potential [41]. Importantly, other studies have also highlighted the involvement of RhoA signalling in the pathogenesis of DMD by contributing to the accumulation of pro-inflammatory macrophages and muscle calcification [42], the relationship with fibrosis [43], and dysregulation of autophagy [44]. Intriguingly, researchers have demonstrated that the expression profile of miR-133b might change depending on DMD progression. Taetzsch et al. [40] analysed the expression of miR-133b in the tibialis anterior muscle of mdx mice at postnatal days 30 and 60. The authors observed elevated expression of miR-133b at day 30 compared with the control group. Similarly, there was a higher expression on day 60. However, it was reduced compared with the analysis from day 30. Thus, the authors demonstrated that the expression of this myomiR increases in a severe period of the disease compared with the subsequent remission period [40]. Lopez et al. [45] observed reduced expression of miR-133b in human DMD skeletal muscle as well as in 6-month-old animals. Therefore, the initial upregulation of miR-133b noted by Taetzsch et al. [40] might represent a compensatory mechanism that is diminished during the later stages of DMD. Mechanistically, Lopez et al. [45] demonstrated that the expression of miR-133b might be repressed by Smad8, a molecule that transduces signals stimulated by bone morphogenic proteins (BMPs). Importantly, *Smad8* messenger RNA (mRNA) was upregulated in DMD human tissue and the mouse mdx^5cv^ model (Figure 1).

**Figure 2:**
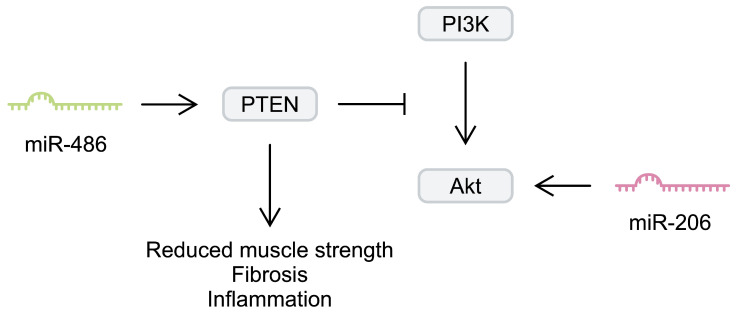
Simplified representation of the impact of miR-206 and miR-489 on the phosphoinositide 3-kinase (PI3K)/Akt signalling pathway.

Another myomiR that has been investigated in the context of DMD is miR-206. Similarly to miR-133b, miR-206 seems to contribute to muscle regeneration. Injecting cardiotoxin into the tibialis anterior muscle of mice induced upregulation of miR-206. Knockdown of miR-206 has been associated with delayed muscle regeneration after injury. To study the impact of miR-206 on DMD, Lu et al. [46] generated mdx mice with miR-206 knockdown. After 4 weeks, animals lacking this myomiR developed significantly more severe dystrophic alterations compared with regular mdx mice. Furthermore, the authors found that miR-206 regulated satellite cell differentiation, as it affected the expression of negative regulators of this process, such as Notch3 [46]. Overexpression of miR-206 in dystrophic tibialis anterior muscle has been associated with upregulation of myogenic regulatory factors, such as RUNX1, MRF4, and MEF-2C. Furthermore, miR-206 stimulated the expression of utrophin A [47]. Upregulation of utrophin A is an important regenerative process in DMD; its elevated expression has been suggested to compensate for the lack of dystrophin [48]. In addition, miR-206 overexpression in dystrophic muscles has been associated with enhanced Akt signalling [47]. Importantly, regulating the Akt pathway has been suggested as a potential beneficial mechanism in DMD animal models [49,50]. Furthermore, dystrophin-deficient myoblasts show enhanced expression of phosphatase and tensin homologue (PTEN), a negative regulator of this signalling pathway [51]. Targeting PTEN could improve dystrophic muscle strength and reduce the necrotic area, fibrosis, and inflammatory infiltration [52]. Additionally, the Akt pathway mediated the pro-myogenic effects and stimulated hypertrophy of myotubes [53]. Therefore, enhancement of Akt signalling could be another beneficial mechanism induced by miRNAs in DMDs. PTEN is targeted by miR-486, a molecule downregulated in murine muscles deficient in dystrophin. Its overexpression in a murine DMD model improved histology (a decreased number of centralised myonuclei and increased myofibre size), reduced CK and ALT serum levels, and enhanced muscle physiology and strength [54] (Figure 2).

Another miRNA downregulated in the skeletal muscles of mdx mice is miR-499-5p. It targets transforming growth factor-β receptors (TGF-βR), thus inhibiting TGF signalling [55]. Decreasing the activity of this pathway seems to be of particular interest due to its promotion of fibrosis. TGF-β levels have been correlated with fibrosis in patients with DMD [56]. Specifically, its presence has been associated with the activity of fibroadipogenic progenitors (FAPs) in mice with severe DMD. Targeting TGF-β has been suggested to reduce muscle degeneration and inflammatory cell infiltration in vivo [57]. Interestingly, reduced levels of miR-499-5p can be rescued by using Wharton’s jelly–derived mesenchymal stem cells (WJ-MSCs). Intravenous administration of WJ-MSCs to mdx mice induced anti-fibrotic effects in the diaphragm [55]. MSCs are self-regenerative cells that have immunomodulatory properties. They secrete cytokines and EVs, which contain bioactive cargo, including miRNAs [58]. Therefore, structures secreted by MSCs can act as nanocarriers that deliver molecules to change the expression profile in targeted cells. For example, co-culture of placenta-derived MSCs (P-MSCs) with myoblasts resulted in the transduction of miR-29c, a molecule that is downregulated in the skeletal muscles of patients with DMD and mdx mice. There were similar results when myoblasts were treated with P-MSC-derived exosomes. Moreover, treatment of DMD myoblasts with these structures could reduce the expression of collagen and TGF-β, demonstrating an anti-fibrotic potential. In an in vivo experiment, treatment of mdx mice with these exosomes was associated with reduced skeletal fibrosis, decreased inflammation, and increased utrophin expression [59].

As mentioned previously, upregulation of utrophin represents a promising DMD treatment. miRNAs target mRNAs and repress their translation. Importantly, utrophin mRNA contains binding sites for miRNAs. Consequently, deletion of these sequences might be associated with elevated expression of utrophin. Indeed, inhibition of miR-196b, let-7c, miR-150, miR-296-5p, and miR-133b binding sites was associated with elevated utrophin expression in human-induced pluripotent stem cells (hiPSCs) induced from the fibroblasts of a patient with DMD. Furthermore, differentiation of these cells towards myotubes was associated with elevated expression of α-sarcoglycan compared with unedited cells [60]. Intriguingly, there was a miR-133b binding site in the utrophin sequence. As previously mentioned, this miRNA acts as a protective factor in the development of DMD. Nevertheless, miRNAs are involved in a broad interaction network and frequently regulate the expression of numerous mRNAs. Therefore, their activity may depend on the cellular context and/or other regulators.

Another alteration that occurs in patients with DMD involves changes in energy metabolism and mitochondrial functionality. Patients with dystrophy seem to have impaired glucose tolerance; their blood glucose levels are significantly elevated in an oral glucose tolerance test (OGTT) compared with controls [61]. Recently, Xu et al. [62] found alterations in several metabolic pathways in patients with DMD, including amino acids, D-glutamine and D-glutamate, and linoleic acid, among others. miRNAs might also be involved in the metabolic dysregulation observed in patients with DMD. Mdx mice with miR-378 deficiency demonstrated improved glucose tolerance after an OGTT. Moreover, there was decreased hepatic expression of interleukin 6 (IL-6) and tumour necrosis factor α (TNFα) in these animals. In addition, a lack of miR-378 changes the expression of several genes associated with lipid metabolism [63]. There was dysregulation of energy metabolism, which may result from impairment of mitochondria in DMD, impaired resting adenosine triphosphate (ATP) production, complex-I-driven respiration, as well as functionality of the citric acid cycle [64]. Dysregulated mitochondrial functionality is also correlated with miRNAs. Specifically, Hong et al. [65] demonstrated that mitochondrial genes are regulated by miRNAs located in the DLK1-Dio3 cluster, known as DD-miRNAs. The authors demonstrated transcriptional similarities between the mdx diaphragm and muscles overexpressing DD-miRNAs, thus suggesting their involvement in the pathogenesis of the disease.

Muscular damage is reversed by a group of muscle stem cells known as satellite cells. They possess self-renewing features and can generate myoblasts. Nevertheless, they lose their regenerative properties during the progression of DMD [66]. Investigating the miRNA expression profile of satellite cells demonstrated that DMD alters the expression of these molecules. In satellite cells obtained from mdx mouse models, miR-1 and miR-206 were found to be upregulated [67]. Perhaps the observed upregulation might indicate activation or regenerative processes, as knockdown of miR-206 is associated with delayed differentiation. Specifically, miR-206 deficiency suppresses elongation and fusion of satellite cells, as well as reducing the expression of myosin heavy chain, a differentiation marker [46]. Additionally, miR-1, which was found upregulated in the previously mentioned study, also enhances satellite cell differentiation and suppresses their proliferation through targeting Pax7 [68]. MiR-27b was also found to regulate the expression of Pax3, thus mediating myogenic differentiation as well [69]. Intriguingly, the differentiation, proliferation, and viability of muscle satellite cells are also regulated by miRNAs. For example, the activity of these cells is mediated by miR-381 [70], miR-378 [71], and miR-377 [72], among many miRNAs. Perhaps manipulation of miRNAs that regulate the viability and functionality of satellite cells could enhance dystrophic muscle regeneration.

### 3.2. MicroRNAs Regulating DMD-Associated Cardiomyopathy

Dystrophin is also present in cardiac muscle, so its deficiency in DMD also affects the heart. The incidence of DMD-associated cardiomyopathy rapidly increases with age and is challenging to diagnose. The pathophysiology of dystrophin-deficient cardiomyopathy involves cardiac fibrosis and progressive left ventricle (LV) dysfunction, which ultimately leads to heart failure [73]. Similarly to skeletal muscle, a lack of dystrophin disrupts its connections between the cytoskeleton and the extracellular matrix, which induces cardiomyocyte degeneration [74]. Recent studies have investigated pathogenetic mechanisms that are associated with the development of cardiomyopathy. For example, calcium overload has been suggested as an important aspect of myocardial remodelling and fibrosis [75]. Importantly, dysregulated miRNAs could also contribute to the development of DMD-associated cardiomyopathy. Using DMD cardiomyocytes obtained from iPSCs, Gartz et al. [76] found that the expression of miR-485-3p, miR-338-3p, let-7b-5p, and miR-298 was reduced in exosomes obtained from dystrophin-deficient cells. By contrast, miR-98-5p, mir-431, miR-346, miR-135b-5p, miR-124-3p, and miR-339-5p were upregulated in DMD cardiac cells compared with controls. Importantly, the introduction of dystrophin could normalise the expression of these molecules. miR-339-5p was greatly overexpressed in DMD exosomes, and the authors analysed its effect on cardiomyocytes. Specifically, exposure to miR-339-5p-depleted EVs could decrease stress-induced cell death and improve the mitochondrial membrane potential [76]. Therefore, this study demonstrated that DMD-cardiomyocytes can secrete exosomes with dysregulated miRNAs that alter the behaviour of other cardiac cells, making them more susceptible to damage.

Another miRNA involved in the functionality of the heart is miR-448-3p. Its downregulation induces cardiac remodelling and promotes fibrosis. In dystrophic hearts, there was reduced expression of miR-448. Mechanistically, it was found to target *Ncf1*, a subunit of nicotinamide adenine dinucleotide oxidase 2 (NOX2), an enzyme associated with ROS [77]. Gonzalez et al. [78] found that NOX2 protein expression is increased fivefold in mdx hearts. Furthermore, the authors revealed elevated levels of ROS in mdx hearts, and the use of NOX inhibitors could reduce ROS generation. Consequently, miR-448 seems to mediate cardioprotection from oxidative stress that is implicated in cardiac impairment in DMD-related cardiomyopathy.

As we mentioned previously, the DMD pathogenesis involves calcium overload. Sarcoplasmic reticulum ATPase 2a (SERCA2a) is an enzyme responsible for calcium reuptake in cardiac cells. Its expression is downregulated in DMD animal models. Interestingly, it is regulated by miR-25, a molecule upregulated in DMD. Introduction of the decoy miR-25 through adenovirus was associated with improved myocyte contractility, fractional shortening, and prolonged survival in mdx/utrn (+/−) mice. Mechanistically, suppression of miR-25 could suppress the activity of the mitogen-activated protein kinase (MAPK) pathway [79]. The miRNAs that are involved in the pathogenesis of DMD are summarised in Table 1.

## 4. MicroRNA Alterations in Duchenne Muscular Dystrophy

In the previous section, we discussed the involvement of miRNAs in the pathogenesis of DMD. However, several studies have demonstrated alterations in circulating levels of miRNAs in DMD patients. These alterations could result from miRNA secretion or release due to muscle degeneration, which has been observed in dystrophic muscles [80]. Interestingly, the levels of the circulating miRNAs might also correlate with disease stage. Perhaps muscle activity is required to regulate the secretion of myomiRs, and terminal stages of the disease could suppress the release of RNA molecules, which was suggested in a study investigating circulatory miRNAs in amyotrophic lateral sclerosis [81]. In this section, we will discuss the relevant studies by dividing them as follows: (i) the diagnostic potential of miRNAs; (ii) miRNA levels in ambulant versus non-ambulant patients; and (iii) the role of miRNAs in detecting carriers. Table 2 provides more detailed data regarding the studies we discuss below.

### 4.1. Diagnostic Potential of microRNAs

In the clinical part of their study, Greco et al. [82] compared the expression of several miRNAs between patients with DMD and age- and sex-matched controls. They assessed them with quadriceps femoris biopsies. miR-31, miR-43c, miR-206, miR-222, miR-223, miR-335, miR-449, and miR-494 were increased, while miR-1, miR-29c, and miR-135a were decreased in patients with DMD compared with controls. Jeanson-Leh et al. [83] investigated a small group of patients with DMD. They found that miR-95, miR-208b, and miR-499 were upregulated, while miR-539 was downregulated in the serum of patients with DMD compared with healthy controls. Moreover, they showed decreased expression of miR-499 in the muscles of patients with DMD. Coenen-Stass et al. [84] found that miR-1a-3p, miR-133a-3p, miR-206-3p, and miR-483-5p were higher in patients with DMD than in controls. Moreover, the ROC (receiver operating characteristic) curve analysis showed that the above miRNAs could effectively discriminate between patients with DMD and healthy people. Trifunov et al. [85] performed a longitudinal study assessing the levels of miR-181a-5p, miR-30c-5p, and miR-206. They showed that miR-30c-5p and miR-206 levels were higher in patients with DMD compared with healthy controls over the entire study length (at three timepoints over 4 years). Moreover, ROC curve analysis showed that miR-206 could be useful in differentiating between patients with DMD and patients with BMD. García-Giménez et al. [86] reported upregulation of miR-122-5p, miR-192-5p, miR-19b-3p, miR-323b-3p, and miR-206 in patients with DMD compared with controls. ROC curve analysis showed strong performance for all miRNAs in differentiating between patients with DMD and healthy individuals: each miRNA presented an area under the curve (AUC) of >0.9. Cacchiarelli et al. [87] investigated miR-1, miR-133, miR-206, and miR-233 serum levels in patients with DMD and their role as potential biomarkers. miR-1, miR-133, and miR-206 were upregulated in patients with DMD compared with healthy controls. Moreover, all three miRNAs were able to discriminate patients with DMD from healthy controls and patients with BMD with very high sensitivity and specificity. Further, all three miRNAs were inversely correlated with the North Star Ambulatory Assessment (NSAA) score, showing that disease progression is associated with an increase in these miRNA levels. Zaharieva et al. [88] found that patients with DMD had higher levels of miR-1, miR-206, miR-31, and miR-133b compared with healthy controls. miR-1, miR-206, miR-31, miR-133a, and miR-133b expression was higher in ambulant patients with DMD than in non-ambulant patients with DMD. Contrary to Cacchiarelli et al. [42], the authors found no correlation between these miRNAs and the NSAA scores. However, there was a positive correlation between miR-1, miR-133b, and forced vital capacity (FVC) values [88]. Hu et al. [89] confirmed miR-1, miR-133, and miR-206 upregulation in patients with DMD compared with healthy controls. Moreover, they found that these miRNAs correlated inversely with clinical factors: muscle strength, muscle function, and quality of life. ROC analysis revealed that all three miRNAs could discriminate between patients with DMD and healthy individuals. Li et al. [90] observed increased miR-1, miR-133, miR-206, miR-208a, miR-208b, and miR-499 expression in patients with DMD compared with healthy individuals. In the ROC analysis, the authors found the ability of all studied miRNAs to differentiate patients with DMD from healthy controls, and the ability of miR-133, miR-206, miR-208b, and miR-499 to differentiate patients with DMD from patients with BMD. Importantly, serum creatine kinase (CK) could not discriminate patients with DMD from patients with BMD. Finally, there was a positive correlation between miR-206, miR-208b, and miR-499 and both age and type IIc muscle fibre content in patients with DMD, indicating the potential role of these miRNAs in assessing disease severity and progression [90]. Llano-Diez et al. [91] measured miR-30c-5p and miR-181a-5p expression levels using a technique called digital droplet polymerase chain reaction (ddPCR). Both miRNAs were elevated in patients with DMD compared with control individuals. Nevertheless, they did not find a statistically significant correlation between these miRNA levels and the NSAA scores. Meng et al. [92] found that the levels of miR-1, miR-133a, miR-133b, miR-206, miR-208a, miR-208b, and miR-499 were higher in patients with DMD than in healthy individuals. In ROC curve analysis, all miRNAs had an AUC > 0.747 for discriminating patients with DMD from healthy people. The authors observed a positive correlation between all miRNAs and lower limb distal muscle strength and a negative correlation between miR-499, miR-208b, miR-133a, miR-133b, and Gowers’ time, which may be valuable in the assessment of disease severity.

### 4.2. MicroRNA Levels in Ambulant versus Non-Ambulant Patients

Catapano et al. [93] compared the levels of miR-21-5p, miR-22-3p, miR-23b-3p, miR-29c-3p, and miR-103a-3p in urinary exosomes between patients with DMD and healthy individuals. Patients with DMD were further divided into two groups: (i) ambulant and (ii) non-ambulant. The authors found that miR-29c-3p levels decreased overall in all patients with DMD compared with healthy controls and in ambulant patients with DMD compared with healthy controls. On the other hand, miR-21-5p and miR-23b-3p decreased in non-ambulant patients with DMD compared with healthy controls [93]. The same group also assessed both free-circulating miRNAs as well as miRNAs derived from extracellular vesicles (EVs) in serum [94]. Regarding the free-circulating miRNAs, they found upregulation of miR-1-3p, miR-133a-3p, and miR-29c-3p in patients with DMD compared with healthy controls. Further analysis after dividing patients with DMD into the ambulant and non-ambulant groups revealed other expression differences. Regarding EV-derived miRNAs, miR-133a-3p was increased while miR-29c-3p was decreased in patients with DMD compared with healthy individuals. Almeida-Becerril et al. [95] compared the levels of several miRNAs between ambulant and non-ambulant patients with DMD. They found that miR-133a-3p, miR-206, miR-21-5p, miR-31-5p, miR-128-3p, and miR-144-3p levels were higher in the ambulant group. The authors observed a negative correlation between these miRNA levels and the Vignos scale score. Other scales assessing muscular function also showed correlations with some of the investigated miRNAs. Moreover, all the above-mentioned miRNAs and miR-1-3p positively correlated with alanine transaminase (ALT), aspartate transaminase (AST), and CK values.

### 4.3. The Role of microRNAs in Detecting Carriers

Anaya-Segura et al. [96] assessed whether miR-206 could be used to differentiate between DMD carriers and healthy women. First, they found that miR-206 levels were higher in DMD carriers. Further, the ROC curve analysis showed that miR-206 levels were able to discriminate DMD carriers from healthy women with high sensitivity (78.26%) and specificity (70.83%). Mousa et al. [97] studied patients with DMD and their families, including the mothers of patients with DMD (DMD carriers). They observed the upregulation of miR-499 and miR-103a-3p as well as the downregulation of miR-208a, miR-103a-5p, miR-206, and miR-191-5p in patients with DMD compared with healthy controls. The same deregulation pattern was observed in DMD carriers compared with healthy individuals. ROC curve analysis showed the perfect ability of miR-499 to discriminate patients with DMD from healthy individuals and DMD carriers from healthy individuals, with sensitivity and specificity equal to 100%. miR-206 and miR-191-5p were also good at differentiating between those groups, but they performed worse than miR-499 [97]. Zhang et al. [98] investigated the levels of miR-1, miR-133a, miR-133b, miR-206, miR-208a, miR-208b, and miR-499 in DMD carriers. All miRNAs were upregulated in DMD carriers compared with control women. ROC curve analysis of the ability to discriminate DMD carriers from healthy controls revealed that all miRNAs showed an AUC > 0.600, with only miR-208a not showing statistical significance. The combination of all seven miRNAs presented an AUC of 0.872, higher than the AUC for CK [98]. Therefore, these studies suggest that miRNAs demonstrate promising diagnostic potential. However, it is important to combine knowledge about the expression of miRNAs in the muscles with their circulating levels to understand the mechanisms occurring in dystrophic muscles. For instance, higher expression of miR-206 in the muscles might suggest that regenerative mechanisms have been initiated in the skeletal tissue. By releasing miR-206 into circulation, this phenomenon occurring in the muscles could be detected.

## 5. Conclusions and Future Perspectives

DMD is a disease with a complex pathology. The current available evidence has demonstrated that miRNAs are dysregulated in patients with DMD and in DMD animal models. Damage to muscle cells may be responsible for elevated levels of certain muscle-specific miRNAs observed in the blood. Importantly, dysregulated molecules could be implemented in the DMD diagnostic process, as several studies have demonstrated that they could discriminate between patients and healthy controls. Moreover, miRNAs have been suggested to discriminate between patients with DMD and patients with BMD. Perhaps monitoring a specific miRNA profile could enhance the diagnosis of DMD in the early stages of the disease or point towards a specific dystrophic diagnosis. In addition, in the future, miRNAs may be implemented to monitor disease progression and indirectly suggest muscle conditions. Furthermore, these molecules seem to be involved in the pathogenesis of DMD. By regulating gene expression, their downregulation may be associated with the overexpression of genes that are involved in muscle fibrosis or inflammatory infiltration. Conflicting results have been published regarding certain molecules, which could result from different animal models or different stages of the disease at the time of analysis. miRNAs regulate signalling pathways, muscle strength, mitochondrial functionality, and cardioprotection. Therefore, manipulation of miRNA expression could represent an interesting approach to treating DMD. This could be achieved by using MSCs. Specifically, gene transfection may allow for the isolation of miRNA-overexpressing exosomes, which could serve as drug nanocarriers. Future studies need to examine methods to successfully manipulate miRNA expression or EV secretion. For example, a recent study demonstrated that stimulation of fibro-adipogenic progenitors with a histone deacetylase inhibitor could increase the secretion of miR-206 in EVs, which could provide beneficial effects in dystrophic muscles [99]. Therefore, pharmacological interventions may also be associated with altered miRNA secretion and gene expression. Additionally, apart from miRNAs, there are other classes of ncRNAs that are involved in the regulation of gene expression, including lncRNAs. There is limited information about the role of these molecules in DMD, and future studies should explore their influence on dystrophic muscles and hearts. lncRNAs interact with a wide range of miRNAs. For example, X-inactive-specific transcript RNA (XIST), the most significantly downregulated molecule in one study, was predicted to interact with 27 miRNAs [100]. Another lncRNA, H19, interacts with dystrophin, thus suppressing its degradation [101]. Understanding these mechanisms might result in the development of novel, targeted therapies in the future.

## Figures and Tables

**Table 1:** Summary of microRNA (miRNA) dysregulation that has been suggested to contribute to the pathogenesis of Duchenne muscular dystrophy (DMD).

Molecule	Mechanism	Reference
miR-133b.	Deletion of miR-133b was associated with reduced cross-sectional area, fewer satellite cells, and decreased muscle regenerative capabilities.	[40]
miR-206.	Knockdown of miR-206 in mdx mice further exacerbated dystrophic alterations.	[46]
	Overexpression of miR-206 enhanced the expression of myogenic regulatory factors and utrophin A.	[47]
miR-486	Overexpression of miR-486 in dystrophin-deficient murine models improved muscle histology, strength, and physiology.	[54]
miR-499-5p	Administration of Wharton’s jelly mesenchymal stem cells into mdx mice increase the expression of miR-499-5p, which has anti-fibrotic effects.	[55]
miR-29c	Exosomes from placenta-derived mesenchymal stem cells can transfer miR-29c to myoblasts, thus reducing fibrosis and inflammation.	[59]
miR-378	Mdx mice lacking miR-378 demonstrated improved glucose tolerance, reduced expression of inflammatory mediators in the liver, and altered expression of lipid metabolism–related genes.	[63]
miR-339-5p	DMD-derived cardiomyocytes secrete exosomes containing miR-339-5p that impairs the response of other cardiac cells to stress.	[76]
miR-448-3p	Downregulation of miR-448-3p, which is observed in dystrophic hearts, was associated with the induction of fibrosis and cardiac remodelling.	[77]
miR-25	Suppression of overexpressed miR-25 in DMD animal models was associated with improved myocyte contractility and enhanced survival.	[79]

DMD: Duchenne muscular dystrophy; miRNA: microRNA; WJ-MSC: Wharton’s jelly mesenchymal stem cells.

**Table 2:** Summary of recent studies regarding microRNAs (miRNAs) in patients with Duchenne muscular dystrophy patients (ordered by their appearance in the text).

Ref.	Year	Population	Comparison	Assessed miRNAs	Outcome	Methodology
[82]	2009	12 patients with DMD	10 age- and sex-matched controls	miR-1, miR-29c, miR-124a, miR-135a, miR-516-3p, miR-31, miR-43c, miR-206, miR-222, miR-223, miR-335, miR-449, and miR-494	↑ miR-31, miR-43c, miR-206, miR-222, miR-223, miR-335, miR-449, and miR-494 in patients with DMD ↓ miR-1, miR-29c, and miR-135a in patients with DMD	miRNAs in human quadriceps femoris biopsies by qPCR
[83]	2014	5 patients with DMD (serum) 3 patients with DMD (muscle biopsy)	3 age-matched controls	miR-95, miR-208a, miR-208b, miR-410, miR-433, miR-494, miR-495, miR-499, and miR-539	↑ miR-95, miR-208b, and miR-499 and ↓ miR-539 in serum of patients with DMD ↓ miR-499 in muscle of patients with DMD	miRNAs in serum and paravertebral/dorsal muscle biopsies by qRT-PCR
[84]	2018	28 patients with DMD	16 HCs	miR-1a-3p, miR-133a-3p, miR-206-3p, miR-483-5p, and miR-483-3p	↑ miR-1a-3p, miR-133a-3p, miR-206-3p, and miR-483-5p in patients with DMD ROC analysis showed significant values for DMD vs HCs (AUC)—miR-1a-3p: (0.980); miR-133a-3p: (0.915); miR-206-3p: (0.998); miR-483-5p: (0.819); miR-483-3p: (0.531)	miRNAs in serum by qRT-PCR
[85]	2020	13 patients with DMD	13 HCs	miR-181a-5p, miR-30c-5p, and miR-206	↑ miR-30c-5p and miR-206 in patients with DMD over the entire study length ROC analysis DMD vs BMD (AUC)—miR-206: (0.82), (0.95), and (0.75) at the first, second, and third timepoints, respectively	miRNAs in serum by ddPCR at the three timepoints over 4 years
[86]	2022	5 patients with DMD	5 HCs	miR-122-5p, miR-192-5p, miR-19b-3p, miR-323b-3p, and miR-206	↑ miR-122-5p, miR-192-5p, miR-19b-3p, miR-323b-3p, and miR-206 in patients with DMD ROC analysis DMD vs HCs—AUC > 0.9 for all miRNAs	miRNAs in plasma by qPCR
[87]	2011	26 patients with DMD	7 HCs	miR-1, miR-133, miR-206, and miR-233	↑ miR-1, miR-133, and miR-206 in patients with DMD ROC analysis DMD vs. BMD and DMD vs. HCs– miR-206: AUC always > 0.94, *p* < 0.001 miR-1: AUC always > 0.84, *p* < 0.01 miR-133: AUC always > 0.76, *p* < 0.01 Inverse correlation between miR-1, miR-133, and miR-206 and NSAA scores	miRNAs in serum by qRT-PCR
[88]	2013	44 patients with DMD	14 HCs	miR-1, miR-206, miR-31, miR-133a, and miR-133b	↑ miR-1, miR-206, miR-31, and miR-133b in patients with DMD ↑ miR-1, miR-206, miR-31, miR-133a, and miR-133b in ambulant patients with DMD compared with non-ambulant patients with DMD No correlation between miRNAs and NSAA scores Positive correlation between miR-1 and miR-133b and FVC values	miRNAs in serum by qRT-PCR
[89]	2014	39 patients with DMD	36 HCs	miR-1, miR-133, and miR-206	↑ miR-1, miR-133b, and miR-206 in patients with DMD Inverse correlations between miR-1 and miR-206 and muscle strength; miR-1, miR-133, and miR-206 and muscle function; miR-1, miR-133, and miR-206 and QoL ROC analysis DMD vs HCs (AUC)—miR-1: (0.93); miR-133: (0.90); miR-206: (0.96)	miRNAs in serum by qRT-PCR
[90]	2014	52 patients with DMD	23 HCs	miR-1, miR-133, miR-206, miR-208a, miR-208b, and miR-499	↑ miR-1, miR-133, miR-206, miR-208a, miR-208b, and miR-499 in patients with DMD ROC analysis showed significant values for DMD vs HCs (AUC), DMD vs BMD [AUC]—miR-1: (0.8227); miR-133: (0.8119), [0.6756]; miR-206: (0.9156), [0.7090]; miR-208a: (0.8127); miR-208b: (0.9323), [0.7115]; miR-499: (0.9900), [0.6987] Positive correlation between miR-206, miR-208b, and miR-499 and both age and type IIc muscle fibre content in patients with DMD	miRNAs in serum by qRT-PCR
[91]	2017	21 patients with DMD	22 age-matched HCs	miR-30c-5p and miR-181a-5p	↑ miR-30c-5p and miR-181a-5p in patients with DMD No correlation between miR-30c-5p and miR-181a-5p and NSAA scores	miRNAs in serum by ddPCR
[92]	2022	48 patients with DMD	53 HCs	miR-1, miR-133a, miR-133b, miR-206, miR-208a, miR-208b, and miR-499	↑ miR-1, miR-133a, miR-133b, miR-206, miR-208a, miR-208b, and miR-499 in patients with DMD ROC analysis DMD vs HCs—AUC > 0.747 for all miRNAs Positive correlation between all miRNAs and lower limb distal muscle strength Negative correlation between miR-499, miR-208b, miR-133a, and miR-133b and Gowers’ time	miRNAs in serum by qPCR
[93]	2018	54 patients with DMD: ambulant (*n* = 31) non-ambulant (*n* = 23)	20 age-matched HCs	miR-21-5p, miR-22-3p, miR-23b-3p, miR-29c-3p and miR-103a-3p	↓ miR-29c-3p in patients with DMD compared with HCs and in ambulant patients with DMD compared with HCs ↓ miR-21-5p and miR-23b-3p in non-ambulant patients with DMD compared with HCs	miRNAs in urinary exosomes by qPCR
[94]	2020	28 patients with DMD: ambulant (*n* = 15) non-ambulant (*n* = 13)	8 HCs	miR-1-3p, miR-133a-3p, miR-133b, miR-200c-3p, miR-660-5p, miR-29c-3p and miR-136-5p	↑ miR-1-3p, miR-133a-3p, and miR-29c-3p in patients with DMD compared with HCs ↑ miR-1-3p, miR-133a-3p, miR-133b and miR-136-5p in ambulant patients with DMD compared with HCs ↑ miR-133a-3p in non-ambulant patients with DMD compared with HCs ↑ miR-660-5p and miR-29c-3p in ambulant patients with DMD compared with non-ambulant patients with DMD	Free circulating miRNAs in serum by qRT-PCR
		16 patients with DMD: ambulant (*n* = 8) non-ambulant (*n* = 8)	8 HCs	miR-1-3p, miR-133a-3p, miR-133b, miR-199a-5p, miR-33a-5p, miR-660-5p, and miR-29c-3p	↑ miR-133a-3p and ↓ miR-29c-3p in patients with DMD compared with HCs ↑ miR-133a-3p and miR-199a-5p in ambulant patients with DMD compared with HCs ↑ miR-133a-3p in non-ambulant patients with DMD compared with HCs	EV-derived miRNAs in serum by qRT-PCR
[95]	2022	28 patients with DMD: ambulant (*n* = 18) non-ambulant (*n* = 6)	miRNA correlation with muscle injury and circulating metabolic parameters	miR-1-3p, miR-133a-3p, miR-206, miR-21-5p, miR-31-5p, miR-128-3p, and miR-144-3p	↑ miR-133a-3p, miR-206, miR-21-5p, miR-31-5p, miR-128-3p, and miR-144-3p in ambulant compared with non-ambulant patients with DMD Positive correlation between all miRNAs and ALT, AST, and CK Negative correlation between miR-133a-3p, miR-206, miR-21-5p, miR-31-5p, miR-128-3p, and miR-144-3p and the Vignos scale score	miRNAs in serum by qRT-PCR
[96]	2016	23 DMD carriers	24 control women	miR-206	↑ miR-206 in DMD carriers ROC analysis for DMD carriers vs control women (AUC)—miR-206: (0.803), *p* < 0.0001	miRNAs in serum by qRT-PCR
[97]	2020	29 patients with DMD 29 DMD carriers	10 HCs for each group	miR-499, miR-103a-3p, miR-223, miR-208a, miR-103a-5p, miR-206 and miR-191-5p	↑ miR-499, miR-103a-3p and ↓ miR-208a, miR-103a-5p, miR-206, and miR-191-5p in patients with DMD compared with HCs ↑ miR-499 and miR-103a-3p and ↓ miR-208a, miR-103a-5p, miR-206, and miR-191-5p in DMD carriers compared with HCs ROC analysis showed significant values for patients with DMD vs HCs (AUC) and DMD carriers vs HCs [AUC]—miR-499: (1.000), [1.000]; miR-206: (0.887), [0.918]; miR-191-5p: (0.887), [0.932]	miRNAs in plasma by qPCR
[98]	2020	34 DMD carriers	33 control women	miR-1, miR-133a, miR-133b, miR-206, miR-208a, miR-208b, and miR-499	↑ miR-1, miR-133a, miR-133b, miR-206, miR-208a, miR-208b, and miR-499 in DMD carriers ROC analysis showed significant values for DMD carriers vs control women (AUC)—miR-1: (0.771); miR-133a: (0.701); miR-133b: (0.779); miR-206: (0.655); miR-208b: (0.730), and miR-499 (0.786)	miRNAs in serum by qRT-PCR

↑, increased; ↓, decreased; ALT, alanine transaminase; AST, aspartate transaminase; AUC, area under the receiver operating characteristic curve; BMD, Becker muscular dystrophy; ddPCR, droplet digital polymerase chain reaction; DMD, Duchenne muscular dystrophy; EVs, extracellular vesicles; FVC, forced vital capacity; HCs, healthy controls; NSAA, North Star Ambulatory Assessment; QoL, quality of life; qPCR, quantitative polymerase chain reaction; qRT-PCT, quantitative reverse transcription polymerase chain reaction; ref., reference.

## Data Availability

Not applicable.

## References

[1] Bez Batti Angulski A., Hosny N., Cohen H., Martin A.A., Hahn D., Bauer J., Metzger J.M. (2023). Duchenne muscular dystrophy: Disease mechanism and therapeutic strategies. Front. Physiol..

[2] Duan D., Goemans N., Takeda S., Mercuri E., Aartsma-Rus A. (2021). Duchenne muscular dystrophy. Nat. Rev. Dis. Primers.

[3] Suthar R., Sankhyan N. (2018). Duchenne Muscular Dystrophy: A Practice Update. Indian J. Pediatr..

[4] Birnkrant D.J., Bushby K., Bann C.M., Apkon S.D., Blackwell A., Colvin M.K., Cripe L., Herron A.R., Kennedy A., Kinnett K. (2018). Diagnosis and management of Duchenne muscular dystrophy, part 3: Primary care, emergency management, psychosocial care, and transitions of care across the lifespan. Lancet Neurol..

[5] Broomfield J., Hill M., Guglieri M., Crowther M., Abrams K. (2021). Life Expectancy in Duchenne Muscular Dystrophy: Reproduced Individual Patient Data Meta-Analysis. Neurology.

[6] Molinaro M., Torrente Y., Villa C., Farini A. (2024). Advancing Biomarker Discovery and Therapeutic Targets in Duchenne Muscular Dystrophy: A Comprehensive Review. Int. J. Mol. Sci..

[7] Chang C., Xu L., Zhang R., Jin Y., Jiang P., Wei K., Shi Y., Zhao J., Xiong M., Guo S. (2022). MicroRNA-Mediated Epigenetic Regulation of Rheumatoid Arthritis Susceptibility and Pathogenesis. Front. Immunol..

[8] Krauze A., Procyk G., Gąsecka A., Garstka-Pacak I., Wrzosek M. (2023). The Role of MicroRNAs in Aortic Stenosis-Lessons from Recent Clinical Research Studies. Int. J. Mol. Sci..

[9] Kiełbowski K., Ptaszyński K., Wójcik J., Wojtyś M.E. (2023). The role of selected non-coding RNAs in the biology of non-small cell lung cancer. Adv. Med. Sci..

[10] Procyk G., Klimczak-Tomaniak D., Sygitowicz G., Tomaniak M. (2022). Circulating and Platelet MicroRNAs in Cardiovascular Risk Assessment and Antiplatelet Therapy Monitoring. J. Clin. Med..

[11] Procyk G., Grodzka O., Procyk M., Gąsecka A., Głuszek K., Wrzosek M. (2023). MicroRNAs in Myocarditis—Review of the Preclinical In Vivo Trials. Biomedicines.

[12] Siracusa J., Koulmann N., Banzet S. (2018). Circulating myomiRs: A new class of biomarkers to monitor skeletal muscle in physiology and medicine. J. Cachexia Sarcopenia Muscle.

[13] Maggio I., Chen X., Gonçalves M.A. (2016). The emerging role of viral vectors as vehicles for DMD gene editing. Genome Med..

[14] Carter J.C., Sheehan D.W., Prochoroff A., Birnkrant D.J. (2018). Muscular Dystrophies. Clin. Chest Med..

[15] Vincik L.Y., Dautel A.D., Staples A.A., Lauck L.V., Armstrong C.J., Howard J.T., McGregor D., Ahmadzadeh S., Shekoohi S., Kaye A.D. (2024). Evolving Role of Viltolarsen for Treatment of Duchenne Muscular Dystrophy. Adv. Ther..

[16] Pilgram G.S., Potikanond S., Baines R.A., Fradkin L.G., Noordermeer J.N. (2010). The roles of the dystrophin-associated glycoprotein complex at the synapse. Mol. Neurobiol..

[17] Huard J., Côté P.Y., Parent A., Bouchard J.P., Tremblay J.P. (1992). Dystrophin-like immunoreactivity in monkey and human brain areas involved in learning and motor functions. Neurosci. Lett..

[18] Keegan N.P. (2020). Pseudoexons of the DMD Gene. J. Neuromuscul. Dis..

[19] Ervasti J.M. (2007). Dystrophin, its interactions with other proteins, and implications for muscular dystrophy. Biochim. Biophys. Acta.

[20] Gao Q.Q., McNally E.M. (2015). The Dystrophin Complex: Structure, Function, and Implications for Therapy. Compr. Physiol..

[21] Le S., Yu M., Hovan L., Zhao Z., Ervasti J., Yan J. (2018). Dystrophin As a Molecular Shock Absorber. ACS Nano.

[22] Batchelor C.L., Winder S.J. (2006). Sparks, signals and shock absorbers: How dystrophin loss causes muscular dystrophy. Trends Cell Biol..

[23] Fortunato F., Rossi R., Falzarano M.S., Ferlini A. (2021). Innovative Therapeutic Approaches for Duchenne Muscular Dystrophy. J. Clin. Med..

[24] Mirouse V. (2023). Evolution and developmental functions of the dystrophin-associated protein complex: Beyond the idea of a muscle-specific cell adhesion complex. Front. Cell Dev. Biol..

[25] Hoffman E.P., Brown R.H., Kunkel L.M. (1987). Dystrophin: The protein product of the Duchenne muscular dystrophy locus. Cell.

[26] Lorin C., Vögeli I., Niggli E. (2015). Dystrophic cardiomyopathy: Role of TRPV2 channels in stretch-induced cell damage. Cardiovasc. Res..

[27] Klingler W., Jurkat-Rott K., Lehmann-Horn F., Schleip R. (2012). The role of fibrosis in Duchenne muscular dystrophy. Acta Myol..

[28] Houang E.M., Sham Y.Y., Bates F.S., Metzger J.M. (2018). Muscle membrane integrity in Duchenne muscular dystrophy: Recent advances in copolymer-based muscle membrane stabilizers. Skelet. Muscle.

[29] Mercuri E., Seferian A.M., Servais L., Deconinck N., Stevenson H., Ni X., Zhang W., East L., Yonren S., Muntoni F. (2023). Safety, tolerability and pharmacokinetics of eteplirsen in young boys aged 6-48 months with Duchenne muscular dystrophy amenable to exon 51 skipping. Neuromuscul. Disord..

[30] Law M.L., Cohen H., Martin A.A., Angulski A.B.B., Metzger J.M. (2020). Dysregulation of Calcium Handling in Duchenne Muscular Dystrophy-Associated Dilated Cardiomyopathy: Mechanisms and Experimental Therapeutic Strategies. J. Clin. Med..

[31] Stedman H.H., Sweeney H.L., Shrager J.B., Maguire H.C., Panettieri R.A., Petrof B., Narusawa M., Leferovich J.M., Sladky J.T., Kelly A.M. (1991). The mdx mouse diaphragm reproduces the degenerative changes of Duchenne muscular dystrophy. Nature.

[32] Aartsma-Rus A., Van Deutekom J.C., Fokkema I.F., Van Ommen G.J., Den Dunnen J.T. (2006). Entries in the Leiden Duchenne muscular dystrophy mutation database: An overview of mutation types and paradoxical cases that confirm the reading-frame rule. Muscle Nerve.

[33] Poyatos-García J., Martí P., Liquori A., Muelas N., Pitarch I., Martinez-Dolz L., Rodríguez B., Gonzalez-Quereda L., Damiá M., Aller E. (2022). Dystrophinopathy Phenotypes and Modifying Factors in DMD Exon 45–55 Deletion. Ann. Neurol..

[34] Monaco A.P., Bertelson C.J., Liechti-Gallati S., Moser H., Kunkel L.M. (1988). An explanation for the phenotypic differences between patients bearing partial deletions of the DMD locus. Genomics.

[35] Flanigan K.M., Dunn D.M., von Niederhausern A., Soltanzadeh P., Gappmaier E., Howard M.T., Sampson J.B., Mendell J.R., Wall C., King W.M. (2009). Mutational spectrum of DMD mutations in dystrophinopathy patients: Application of modern diagnostic techniques to a large cohort. Hum. Mutat..

[36] Aartsma-Rus A., Ginjaar I.B., Bushby K. (2016). The importance of genetic diagnosis for Duchenne muscular dystrophy. J. Med. Genet..

[37] Takeshima Y., Yagi M., Okizuka Y., Awano H., Zhang Z., Yamauchi Y., Nishio H., Matsuo M. (2010). Mutation spectrum of the dystrophin gene in 442 Duchenne/Becker muscular dystrophy cases from one Japanese referral center. J. Hum. Genet..

[38] Sarker S., Eshaque T.B., Soorajkumar A., Nassir N., Zehra B., Kanta S.I., Rahaman M.A., Islam A., Akter S., Ali M.K. (2023). Mutational spectrum and phenotypic variability of Duchenne muscular dystrophy and related disorders in a Bangladeshi population. Sci. Rep..

[39] Iannone F., Montesanto A., Cione E., Crocco P., Caroleo M.C., Dato S., Rose G., Passarino G. (2020). Expression Patterns of Muscle-Specific miR-133b and miR-206 Correlate with Nutritional Status and Sarcopenia. Nutrients.

[40] Taetzsch T., Shapiro D., Eldosougi R., Myers T., Settlage R.E., Valdez G. (2021). The microRNA miR-133b functions to slow Duchenne muscular dystrophy pathogenesis. J. Physiol..

[41] Mu X., Usas A., Tang Y., Lu A., Wang B., Weiss K., Huard J. (2013). RhoA mediates defective stem cell function and heterotopic ossification in dystrophic muscle of mice. FASEB J..

[42] Mu X., Lin C.Y., Hambright W.S., Tang Y., Ravuri S., Lu A., Matre P., Chen W., Gao X., Cui Y. (2020). Aberrant RhoA activation in macrophages increases senescence-associated secretory phenotypes and ectopic calcification in muscular dystrophic mice. Aging.

[43] Fernández-Simón E., Suárez-Calvet X., Carrasco-Rozas A., Piñol-Jurado P., López-Fernández S., Pons G., Bech Serra J.J., de la Torre C., de Luna N., Gallardo E. (2022). RhoA/ROCK2 signalling is enhanced by PDGF-AA in fibro-adipogenic progenitor cells: Implications for Duchenne muscular dystrophy. J. Cachexia Sarcopenia Muscle.

[44] You J.S., Kim Y., Lee S., Bashir R., Chen J. (2023). RhoA/ROCK signalling activated by ARHGEF3 promotes muscle weakness via autophagy in dystrophic mdx mice. J. Cachexia Sarcopenia Muscle.

[45] Lopez M.A., Si Y., Hu X., Williams V., Qushair F., Carlyle J., Alesce L., Conklin M., Gilbert S., Bamman M.M. (2022). Smad8 Is Increased in Duchenne Muscular Dystrophy and Suppresses miR-1, miR-133a, and miR-133b. Int. J. Mol. Sci..

[46] Liu N., Williams A.H., Maxeiner J.M., Bezprozvannaya S., Shelton J.M., Richardson J.A., Bassel-Duby R., Olson E.N. (2012). microRNA-206 promotes skeletal muscle regeneration and delays progression of Duchenne muscular dystrophy in mice. J. Clin. Investig..

[47] Amirouche A., Jahnke V.E., Lunde J.A., Koulmann N., Freyssenet D.G., Jasmin B.J. (2017). Muscle-specific microRNA-206 targets multiple components in dystrophic skeletal muscle representing beneficial adaptations. Am. J. Physiol. Cell Physiol..

[48] Soblechero-Martín P., López-Martínez A., de la Puente-Ovejero L., Vallejo-Illarramendi A., Arechavala-Gomeza V. (2021). Utrophin modulator drugs as potential therapies for Duchenne and Becker muscular dystrophies. Neuropathol. Appl. Neurobiol..

[49] Gurpur P.B., Liu J., Burkin D.J., Kaufman S.J. (2009). Valproic acid activates the PI3K/Akt/mTOR pathway in muscle and ameliorates pathology in a mouse model of Duchenne muscular dystrophy. Am. J. Pathol..

[50] Boppart M.D., Burkin D.J., Kaufman S.J. (2011). Activation of AKT signaling promotes cell growth and survival in α7β1 integrin-mediated alleviation of muscular dystrophy. Biochim. Biophys. Acta.

[51] Yazid M.D., Hung-Chih C. (2021). Perturbation of PI3K/Akt signaling affected autophagy modulation in dystrophin-deficient myoblasts. Cell Commun. Signal..

[52] Yue F., Song C., Huang D., Narayanan N., Qiu J., Jia Z., Yuan Z., Oprescu S.N., Roseguini B.T., Deng M. (2021). PTEN Inhibition Ameliorates Muscle Degeneration and Improves Muscle Function in a Mouse Model of Duchenne Muscular Dystrophy. Mol. Ther..

[53] Go G.Y., Jo A., Seo D.W., Kim W.Y., Kim Y.K., So E.Y., Chen Q., Kang J.S., Bae G.U., Lee S.J. (2020). Ginsenoside Rb1 and Rb2 upregulate Akt/mTOR signaling-mediated muscular hypertrophy and myoblast differentiation. J. Ginseng Res..

[54] Alexander M.S., Casar J.C., Motohashi N., Vieira N.M., Eisenberg I., Marshall J.L., Gasperini M.J., Lek A., Myers J.A., Estrella E.A. (2014). MicroRNA-486-dependent modulation of DOCK3/PTEN/AKT signaling pathways improves muscular dystrophy-associated symptoms. J. Clin. Investig..

[55] Park S.E., Jeong J.B., Oh S.J., Kim S.J., Kim H., Choi A., Choi S.J., Oh S.Y., Ryu G.H., Lee J. (2021). Wharton’s Jelly-Derived Mesenchymal Stem Cells Reduce Fibrosis in a Mouse Model of Duchenne Muscular Dystrophy by Upregulating microRNA 499. Biomedicines.

[56] Ismaeel A., Kim J.S., Kirk J.S., Smith R.S., Bohannon W.T., Koutakis P. (2019). Role of Transforming Growth Factor-β in Skeletal Muscle Fibrosis: A Review. Int. J. Mol. Sci..

[57] Mázala D.A., Novak J.S., Hogarth M.W., Nearing M., Adusumalli P., Tully C.B., Habib N.F., Gordish-Dressman H., Chen Y.W., Jaiswal J.K. (2020). TGF-β-driven muscle degeneration and failed regeneration underlie disease onset in a DMD mouse model. JCI Insight.

[58] Bakinowska E., Kiełbowski K., Pawlik A. (2023). The Role of Extracellular Vesicles in the Pathogenesis and Treatment of Rheumatoid Arthritis and Osteoarthritis. Cells.

[59] Bier A., Berenstein P., Kronfeld N., Morgoulis D., Ziv-Av A., Goldstein H., Kazimirsky G., Cazacu S., Meir R., Popovtzer R. (2018). Placenta-derived mesenchymal stromal cells and their exosomes exert therapeutic effects in Duchenne muscular dystrophy. Biomaterials.

[60] Sengupta K., Mishra M.K., Loro E., Spencer M.J., Pyle A.D., Khurana T.S. (2020). Genome Editing-Mediated Utrophin Upregulation in Duchenne Muscular Dystrophy Stem Cells. Mol. Ther. Nucleic Acids.

[61] Bostock E.L., Edwards B.T., Jacques M.F., Pogson J.T.S., Reeves N.D., Onambele-Pearson G.L., Morse C.I. (2018). Impaired Glucose Tolerance in Adults with Duchenne and Becker Muscular Dystrophy. Nutrients.

[62] Xu H., Cai X., Xu K., Wu Q., Xu B. (2023). The metabolomic plasma profile of patients with Duchenne muscular dystrophy: Providing new evidence for its pathogenesis. Orphanet J. Rare Dis..

[63] Podkalicka P., Mucha O., Kaziród K., Szade K., Stępniewski J., Ivanishchuk L., Hirao H., Pośpiech E., Józkowicz A., Kupiec-Weglinski J.W. (2022). miR-378 affects metabolic disturbances in the mdx model of Duchenne muscular dystrophy. Sci. Rep..

[64] Casati S.R., Cervia D., Roux-Biejat P., Moscheni C., Perrotta C., De Palma C. (2024). Mitochondria and Reactive Oxygen Species: The Therapeutic Balance of Powers for Duchenne Muscular Dystrophy. Cells.

[65] Vu Hong A., Bourg N., Sanatine P., Poupiot J., Charton K., Gicquel E., Massourides E., Spinazzi M., Richard I., Israeli D. (2023). Dlk1-Dio3 cluster miRNAs regulate mitochondrial functions in the dystrophic muscle in Duchenne muscular dystrophy. Life Sci. Alliance.

[66] Cardone N., Taglietti V., Baratto S., Kefi K., Periou B., Gitiaux C., Barnerias C., Lafuste P., Pharm F.L., Pharm J.N. (2023). Myopathologic trajectory in Duchenne muscular dystrophy (DMD) reveals lack of regeneration due to senescence in satellite cells. Acta Neuropathol. Commun..

[67] Pietraszek-Gremplewicz K., Kozakowska M., Bronisz-Budzynska I., Ciesla M., Mucha O., Podkalicka P., Madej M., Glowniak U., Szade K., Stepniewski J. (2018). Heme Oxygenase-1 Influences Satellite Cells and Progression of Duchenne Muscular Dystrophy in Mice. Antioxid. Redox Signal..

[68] Chen J.F., Tao Y., Li J., Deng Z., Yan Z., Xiao X., Wang D.Z. (2010). microRNA-1 and microRNA-206 regulate skeletal muscle satellite cell proliferation and differentiation by repressing Pax7. J. Cell Biol..

[69] Crist C.G., Montarras D., Pallafacchina G., Rocancourt D., Cumano A., Conway S.J., Buckingham M. (2009). Muscle stem cell behavior is modified by microRNA-27 regulation of Pax3 expression. Proc. Natl. Acad. Sci. USA.

[70] Shen J., Wang J., Zhen H., Liu Y., Li L., Luo Y., Hu J., Liu X., Li S., Hao Z. (2022). MicroRNA-381 Regulates Proliferation and Differentiation of Caprine Skeletal Muscle Satellite Cells by Targeting. Int. J. Mol. Sci..

[71] Li H., Kang L., Wu R., Li C., Zhang Q., Zhong R., Jia L., Zhu D., Zhang Y. (2022). miR-378-mediated glycolytic metabolism enriches the Pax7. Cell Regen..

[72] Zhu Y., Li P., Dan X., Kang X., Ma Y., Shi Y. (2022). Inhibits Proliferation and Differentiation of Bovine Skeletal Muscle Satellite Cells by Targeting. Genes.

[73] Kamdar F., Garry D.J. (2016). Dystrophin-Deficient Cardiomyopathy. J. Am. Coll. Cardiol..

[74] Fayssoil A., Nardi O., Orlikowski D., Annane D. (2010). Cardiomyopathy in Duchenne muscular dystrophy: Pathogenesis and therapeutics. Heart Fail. Rev..

[75] Morales E.D., Yue Y., Watkins T.B., Han J., Pan X., Gibson A.M., Hu B., Brito-Estrada O., Yao G., Makarewich C.A. (2023). Dwarf Open Reading Frame (DWORF) Gene Therapy Ameliorated Duchenne Muscular Dystrophy Cardiomyopathy in Aged mdx Mice. J. Am. Heart Assoc..

[76] Gartz M., Beatka M., Prom M.J., Strande J.L., Lawlor M.W. (2021). Cardiomyocyte-produced miR-339-5p mediates pathology in Duchenne muscular dystrophy cardiomyopathy. Hum. Mol. Genet..

[77] Kyrychenko S., Kyrychenko V., Badr M.A., Ikeda Y., Sadoshima J., Shirokova N. (2015). Pivotal role of miR-448 in the development of ROS-induced cardiomyopathy. Cardiovasc. Res..

[78] Gonzalez D.R., Treuer A.V., Lamirault G., Mayo V., Cao Y., Dulce R.A., Hare J.M. (2014). NADPH oxidase-2 inhibition restores contractility and intracellular calcium handling and reduces arrhythmogenicity in dystrophic cardiomyopathy. Am. J. Physiol. Heart Circ. Physiol..

[79] Kepreotis S.V., Oh J.G., Park M., Yoo J., Lee C., Mercola M., Hajjar R.J., Jeong D. (2024). Inhibition of miR-25 ameliorates cardiac and skeletal muscle dysfunction in aged. Mol. Ther. Nucleic Acids.

[80] Abdel-Salam E., Abdel-Meguid I., Korraa S.S. (2009). Markers of degeneration and regeneration in Duchenne muscular dystrophy. Acta Myol..

[81] Toivonen J.M., Manzano R., Oliván S., Zaragoza P., García-Redondo A., Osta R. (2014). MicroRNA-206: A potential circulating biomarker candidate for amyotrophic lateral sclerosis. PLoS ONE.

[82] Greco S., De Simone M., Colussi C., Zaccagnini G., Fasanaro P., Pescatori M., Cardani R., Perbellini R., Isaia E., Sale P. (2009). Common micro-RNA signature in skeletal muscle damage and regeneration induced by Duchenne muscular dystrophy and acute ischemia. FASEB J..

[83] Jeanson-Leh L., Lameth J., Krimi S., Buisset J., Amor F., Le Guiner C., Barthélémy I., Servais L., Blot S., Voit T. (2014). Serum profiling identifies novel muscle miRNA and cardiomyopathy-related miRNA biomarkers in Golden Retriever muscular dystrophy dogs and Duchenne muscular dystrophy patients. Am. J. Pathol..

[84] Coenen-Stass A.M.L., Sork H., Gatto S., Godfrey C., Bhomra A., Krjutškov K., Hart J.R., Westholm J.O., O’Donovan L., Roos A. (2018). Comprehensive RNA-Sequencing Analysis in Serum and Muscle Reveals Novel Small RNA Signatures with Biomarker Potential for DMD. Mol. Ther. Nucleic Acids.

[85] Trifunov S., Natera-de Benito D., Exposito Escudero J.M., Ortez C., Medina J., Cuadras D., Badosa C., Carrera L., Nascimento A., Jimenez-Mallebrera C. (2020). Longitudinal Study of Three microRNAs in Duchenne Muscular Dystrophy and Becker Muscular Dystrophy. Front. Neurol..

[86] García-Giménez J.L., García-Trevijano E.R., Avilés-Alía A.I., Ibañez-Cabellos J.S., Bovea-Marco M., Bas T., Pallardó F.V., Viña J.R., Zaragozá R. (2022). Identification of circulating miRNAs differentially expressed in patients with Limb-girdle, Duchenne or facioscapulohumeral muscular dystrophies. Orphanet J. Rare Dis..

[87] Cacchiarelli D., Legnini I., Martone J., Cazzella V., D’Amico A., Bertini E., Bozzoni I. (2011). miRNAs as serum biomarkers for Duchenne muscular dystrophy. EMBO Mol. Med..

[88] Zaharieva I.T., Calissano M., Scoto M., Preston M., Cirak S., Feng L., Collins J., Kole R., Guglieri M., Straub V. (2013). Dystromirs as serum biomarkers for monitoring the disease severity in Duchenne muscular Dystrophy. PLoS ONE.

[89] Hu J., Kong M., Ye Y., Hong S., Cheng L., Jiang L. (2014). Serum miR-206 and other muscle-specific microRNAs as non-invasive biomarkers for Duchenne muscular dystrophy. J. Neurochem..

[90] Li X., Li Y., Zhao L., Zhang D., Yao X., Zhang H., Wang Y.C., Wang X.Y., Xia H., Yan J. (2014). Circulating Muscle-specific miRNAs in Duchenne Muscular Dystrophy Patients. Mol. Ther. Nucleic Acids.

[91] Llano-Diez M., Ortez C.I., Gay J.A., Álvarez-Cabado L., Jou C., Medina J., Nascimento A., Jimenez-Mallebrera C. (2017). Digital PCR quantification of miR-30c and miR-181a as serum biomarkers for Duchenne muscular dystrophy. Neuromuscul. Disord..

[92] Meng Q., Zhang J., Zhong J., Zeng D., Lan D. (2022). Novel miRNA Biomarkers for Patients With Duchenne Muscular Dystrophy. Front. Neurol..

[93] Catapano F., Domingos J., Perry M., Ricotti V., Phillips L., Servais L., Seferian A., Groot I., Krom Y.D., Niks E.H. (2018). Downregulation of miRNA-29, -23 and -21 in urine of Duchenne muscular dystrophy patients. Epigenomics.

[94] Catapano F., Scaglioni D., Maresh K., Ala P., Domingos J., Selby V., Ricotti V., Phillips L., Servais L., Seferian A. (2020). Novel free-circulating and extracellular vesicle-derived miRNAs dysregulated in Duchenne muscular dystrophy. Epigenomics.

[95] Almeida-Becerril T., Rodríguez-Cruz M., Hernández-Cruz S.Y., Ruiz-Cruz E.D., Mendoza C.R.S., Cárdenas-Conejo A., Escobar-Cedillo R.E., Ávila-Moreno F., Aquino-Jarquin G. (2022). Natural history of circulating miRNAs in Duchenne disease: Association with muscle injury and metabolic parameters. Acta Neurol. Scand..

[96] Anaya-Segura M.A., Rangel-Villalobos H., Martínez-Cortés G., Gómez-Díaz B., Coral-Vázquez R.M., Zamora-González E.O., García S., López-Hernández L.B. (2016). Serum Levels of MicroRNA-206 and Novel Mini-STR Assays for Carrier Detection in Duchenne Muscular Dystrophy. Int. J. Mol. Sci..

[97] Mousa N.O., Abdellatif A., Fahmy N., Zada S., El-Fawal H., Osman A. (2020). Circulating MicroRNAs in Duchenne Muscular Dystrophy. Clin. Neurol. Neurosurg..

[98] Zhang J., Meng Q., Zhong J., Zhang M., Qin X., Ni X., Ma J., He Y., Zeng D., Lan D. (2020). Serum MyomiRs as Biomarkers for Female Carriers of Duchenne/Becker Muscular Dystrophy. Front. Neurol..

[99] Sandonà M., Consalvi S., Tucciarone L., De Bardi M., Scimeca M., Angelini D.F., Buffa V., D’Amico A., Bertini E.S., Cazzaniga S. (2020). HDAC inhibitors tune miRNAs in extracellular vesicles of dystrophic muscle-resident mesenchymal cells. EMBO Rep..

[100] Xu X., Hao Y., Xiong S., He Z. (2020). Comprehensive Analysis of Long Non-coding RNA-Associated Competing Endogenous RNA Network in Duchenne Muscular Dystrophy. Interdiscip. Sci..

[101] Zhang Y., Li Y., Hu Q., Xi Y., Xing Z., Zhang Z., Huang L., Wu J., Liang K., Nguyen T.K. (2020). The lncRNA H19 alleviates muscular dystrophy by stabilizing dystrophin. Nat. Cell Biol..

